# Added Value of Cognition in the Prediction of Survival in Low and High Grade Glioma

**DOI:** 10.3389/fneur.2021.773908

**Published:** 2021-11-18

**Authors:** Emma van Kessel, Ewoud Schuit, Irene M. C. Huenges Wajer, Carla Ruis, Filip Y. F. L. De Vos, Joost J. C. Verhoeff, Tatjana Seute, Martine J. E. van Zandvoort, Pierre A. Robe, Tom J. Snijders

**Affiliations:** ^1^University Medical Center Utrecht/UMC Utrecht Brain Center, Department of Neurology & Neurosurgery, Utrecht University, Utrecht, Netherlands; ^2^Julius Center for Health Sciences and Primary Care, University Medical Center Utrecht, Utrecht University, Utrecht, Netherlands; ^3^Experimental Psychology, Helmholtz Institute, Utrecht University, Utrecht, Netherlands; ^4^University Medical Center Utrecht/UMC Utrecht Brain Center, Department of Medical Oncology, Utrecht University, Utrecht, Netherlands; ^5^University Medical Center Utrecht/UMC Utrecht Brain Center, Department of Radiation Oncology, Utrecht University, Utrecht, Netherlands

**Keywords:** diffuse glioma, cognition, prediction models, added value, prognosis, survival

## Abstract

**Background:** Diffuse gliomas, which are at WHO grade II-IV, are progressive primary brain tumors with great variability in prognosis. Our aim was to investigate whether pre-operative cognitive functioning is of added value in survival prediction in these patients.

**Methods:** In a retrospective cohort study of patients undergoing awake craniotomy between 2010 and 2019 we performed pre-operative neuropsychological assessments in five cognitive domains. Their added prognostic value on top of known prognostic factors was assessed in two patient groups [low- (LGG) and high-grade gliomas (HGG]). We compared Cox proportional hazards regression models with and without the cognitive domain by means of loglikelihood ratios tests (LRT), discriminative performance measures (by AUC), and risk classification [by Integrated Discrimination Index (IDI)].

**Results:** We included 109 LGG and 145 HGG patients with a median survival time of 1,490 and 511 days, respectively. The domain memory had a significant added prognostic value in HGG as indicated by an LRT (*p*-value = 0.018). The cumulative AUC for HGG with memory included was.78 (*SD* = 0.017) and without cognition 0.77 (*SD* = 0.018), IDI was 0.043 (0.000–0.102). In LGG none of the cognitive domains added prognostic value.

**Conclusions:** Our findings indicated that memory deficits, which were revealed with the neuropsychological examination, were of additional prognostic value in HGG to other well-known predictors of survival.

## Introduction

Diffuse gliomas, which are at WHO grade II-IV, are progressive primary brain tumors with a variable, but generally poor prognosis, despite recent progress in treatment options. Until now, research yielded several important predictors of survival, including histomolecular classification, age, the extent of resection, preoperative tumor volume, and Karnofsky performance status (KPS) for both high- and low-grade glioma (HGG and LGG, respectively) (1–4). Additionally, several prognostic factors for specific grades of tumors were reported. For low-grade glioma, the presence of neurologic deficits before surgery (not including epilepsy) and midline crossing are unfavorable predictors. For high-grade glioma, predictors include MGMT promoter methylation status and minimal mental state examination (MMSE) score (2, 5). These prognostic factors are important to personalize treatment and rehabilitation, and to stratify patients for clinical trials. Additionally, identification of certain, molecular or neurocognitive, prognostic markers, can lead to new insights into the pathophysiological mechanisms of diffuse glioma.

Cognitive deficits occur in all different grades of glioma (6, 7). In a recent study, we found these deficits to be independently, and possibly causally, related to survival in diffuse gliomas (8). However, if an independent or causal relationship is demonstrated between a determinant and outcome in such an etiological study, this does not necessarily mean that this variable is of added value to existing prediction models or known prognostic factors for the prediction of survival. Whereas the main goal in etiological research is to demonstrate relationships at a group level, prognostic research focuses on estimating the risk of future events for an individual patient. As such, investigations into the prognostic value of previously demonstrated causally related factors are sensible. In particular, to assess whether such factors have added value on top of existing prediction models or sets of known predictors.

To our knowledge, research in this field has been focused mainly on HGG and no data have been published about cognition as a predictor of survival for diffuse gliomas based on the WHO 2016-classification of Central Nervous System (CNS) tumors (9). In this work, we performed a retrospective cohort study to investigate the added prognostic value of cognitive functioning in treatment-naive patients with diffuse gliomas of all grades (II-IV), in addition to well-recognized predictors of survival in these patients.

## Materials and Methods

### Design and Participants

We performed a single-center retrospective study in a cohort of treatment-naive diffuse glioma patients who underwent elaborate neuropsychological testing as part of their pre-operative work-up for awake brain surgery between January 2010 and July 2019 at the University Medical Center in Utrecht, The Netherlands (UMCU).

Inclusion criteria for this study were the presence of a diffuse glioma according to the criteria of WHO 2016 and a minimum age of 18 years. For tumors diagnosed before 2016, we used all available histological and molecular data (from immunohistochemical staining and targeted next-generation sequencing) from clinical practice to (re-)classify the tumor according to WHO 2016 criteria. Since a small sample (7.9%) (re-)classification was not possible based on the available molecular data, we labeled these as “missing values” and performed imputation later on.

Exclusion criteria were as follows:

(a) Any form of tumor-directed treatment, such as tumor reductive surgery, chemotherapy, and radiotherapy, before neuropsychological assessment. Having undergone a biopsy shortly before a planned resection was allowed. Symptom-directed treatments such as anti-epileptic drugs and dexamethasone were allowed as well.
(b) Incomplete neuropsychological assessment (due to emergency surgery or tumors merely located in the motor strip, for instance). Data were considered complete if more than 50% of tasks within one domain were performed.

Since the various glioma subtypes differ greatly in their biological behavior as well as their prognosis, it is possible that the effect of cognition, as well as other determinants, on survival also differs between WHO 2016 glioma subtypes. For this reason, we performed all analyses separately in HGG (Grade II/III Astrocytoma IDH-Wildtype, Glioblastoma IDH-mutated, and IDH-Wildtype) and LGG (Grade II/III Astrocytoma IDH-mutated, and Grade II/II Oligodendroglioma 1p19q-codeleted) patients.

The UMCU institutional ethical review board approved the study. The informed consent was not obtained for this observational study on data that were obtained as part of routine clinical care (protocol code METC 17/384 and 09-420).

### Neuropsychological Tests

In the study sample, we focused on neurocognitive functioning (NCF) scores for five predefined cognitive domains, namely, attention and executive functioning, memory, psychomotor speed, language, and visuospatial functioning. The neuropsychological instruments that were used as part of our routine clinical care are listed in Table 1. These tests are internationally widely used, standardized psychometric instruments for assessing neurocognitive deficits (although not specific for oncology patients) (10).

**Table 1:** Neuropsychological tasks per domain.

**Attention & Executive Functioning**
Wechsler Adult Intelligence Scale (WAIS) Digit Span Forward^a^ Trail Making Test (TMT) Switching ratio (TMTB/TMTA)^b^ Phonologic Fluency^c^ Stroop/Delis Kaplan Executive Function System (DKEFS) inhibition ratio^d^ Wechsler Adult Intelligence Scale (WAIS_IV) Digit Span Backward
**Memory**
RAVLT-Dutch Version immediate, delay, recognition^e^ Rey-Osterieth Complex Figure Test (ROCF) delay^f^ Semantic Fluency^g^
**Visuospatial functioning**
Judgment of Line Orientation (JULO)^h^ ROCF Copy
**Psychomotor Speed**
Stroop/DKEFS I Stroop/DKEFS II TMTA
**Language**
Boston Naming Test^i^ Token Test^j^

a *Wechsler Adult Intelligence Scale Third Edition Digit Span [WAIS-III] (11), Wechsler Adult Intelligence Scale Fourth Edition Digit Span [WAIS-IV] (12)*.

b *Trail Making Test [TMT] (13)*.

c *Phonologic Verbal Fluency Test [Lexical Fluency] (14, 15)*.

d *Delis-Kaplan Executive Function System [DKEFS] (16)*.

e *15 Words Test [15WT] (17)*.

f *Rey-Osterieth Complex Figure Test [ROCF] (18, 19)*.

g *Semantic Verbal Fluency Test [Semantic Fluency] (14)*.

h *Judgment of Line Orientation [JULO] (20)*.

i *Boston Naming Task [BNT] (21)*.

j *Token Test [TT] (22)*.

Neuropsychological tests often tap into more than one cognitive domain and classification into cognitive domains often varies in the literature. We made use of a predetermined test classification in accordance with previous studies and literature (Table 1) (23–25). The neuropsychological evaluation was conducted shortly (1–7 days) before the awake brain tumor surgery by an experienced clinical neuropsychologist (CR, MvZ, and IHW). Each neuropsychological test was scored according to standardized scoring criteria. For normative comparisons, the unadjusted scores were transformed into *Z*-scores based on the *M* and *SD* of control subjects derived from published norm data. Use of corticosteroids or anticonvulsants at the time of NPA did not serve as grounds for exclusion.

We measured NCF data at the individual patient level, which means that we counted the number of individual patients with an impaired performance per domain. A patient was considered impaired in a given domain if the patient performed below −2 *SD* on any of the administered (sub) tests within that domain, in accordance with previous studies and based on clinical practice (7). We used a threshold of −1.5 *SD* for cognitive deficits in each domain because of the lower frequencies of impairments in LGG. This was an epidemiological choice to increase the variability in the determinant, however, this more liberal threshold was still clinically relevant and was used in several previous studies before (6).

### Data Collection

At our center, all neuropsychological data are prospectively collected. We extracted data on patient characteristics from the electronic patient file for all diffuse glioma patients undergoing awake surgery between 2010 and 2019. Data included sex, age at surgical resection, survival time and status, integrated (‘layered') histomolecular diagnosis based on WHO 2016 classification, extent of resection, O^6^-methylguanine-DNA methyltransferase (MGMT)-methylation status of the tumor, Karnofsky Performance Scale score (KPS), preoperative tumor volume, and neurologic deficits or epileptic seizures at presentation (5, 26). Volumes were measured in 3D with the use of Osirix Lite version 9.5.2: by Pixmeo R version 4.0.3: by RStudio PBC on T2-/fluid-attenuated inversion recovery (FLAIR)-weighted MRI scans and the volume was defined as the whole area of hyperintensity. This represented the total lesion volume, including tumor infiltration and edema. Volumes were measured by a neuro-oncological neurosurgeon and a junior clinical scientist under the supervision of the same neurosurgeon. Since this parameter was independent of enhancement (and thereby grade) of the lesion, it formed a widely usable representation of the extent of brain volume that was potentially hampered in its function by the tumor in any way (27). The extent of resection was based on the surgical report from the electronic patient file and classified into three different categories, which were biopsy or debulking (1–78%), 79–90%, and 91–100% of macroscopically complete (“gross total”) tumor-resection. According to literature, this classification has the highest clinical relevance (28–30). In cases where percentages of resection were not reported, we did not calculate percentages based on the report, but classified “gross total” as 91–100%, “subtotal or incomplete” as 79–90%, and “partially or only small part could be removed” as “1–78%”.

Survival time was defined as the period between the first respective neurosurgery and the date of death from cancer or any other cause or censored at the date of last follow-up (March 1, 2020).

### Statistical Analyses

We established, for all different cognitive domains, the additional value to a model with well-recognized predictors of survival per patient group (see below).

Analyses were performed with R version 4.0.3. First, we assessed missing data and whether data were missing completely at random (MCAR) by means of an MCAR table in which patients without missing values was compared with patients with one or more missing values. In order to avoid bias and a decrease in power due to missing data, we imputed missing values by means of multiple imputations (10 imputation sets). The imputation model included all new and existing predictors as well as the outcome. Results were pooled across imputation sets using Rubin's Rules (31).

We analyzed baseline characteristics with descriptive statistics. Univariable analysis was performed to assess the (unadjusted or crude) association of the five cognitive domains of interest and all other determinants with survival, by univariable Cox proportional hazard (CPH) models.

#### Model Preparation: Schoenfeld, Df-Beta Residuals, and Collinearity

The Cox model assumes that survival curves of two strata follow hazard functions that are proportional over time. This proportional hazard (PH) assumption was checked for all determinants with log-minus-log plots and by Schoenfeld residuals. Scaled Schoenfeld residuals helped to decide whether the proportional hazards assumption holds, in addition to the log-minus-log plots.

We calculated Df Beta residuals to decide which cases were (too) influential in estimating the model parameters. We performed sensitivity analyses by excluding “influential” patients and checked why these patients were of such great influence.

Before performing survival analyses, we tested for multicollinearity between the determinants KPS and all cognitive domains by Pearson correlation coefficients.

Furthermore, the potential non-linearity of the association between continuous predictors and the outcome was assessed using restricted cubic splines.

#### Determining the Additional Prognostic Value

We determined the added prognostic value for all five different cognitive domains. There were several performance measures available for quantifying the added value of predictive variables (32).

We calculated added prognostic values by comparing different measures of goodness of fit and predictive performance of the models with and without cognitive functioning included as a predictor. In both HGG and LGG patients, we used a “baseline model” with known predictors from literature and without the inclusion of cognition. These parameters differed for both patient groups (1, 3). We adhered quite strictly to the prognostic factors that are already used as such in models in the literature. Thereafter, we added each one of the cognitive domains to the model separately: resulting in five models per patient group. For all these models, we used multivariable cox-proportional hazard (CPH) regression analyses. The following measures were compared between the baseline model and the five cognitive domain extended models: loglikelihood [formally tested using a likelihood ratio test (LRT)], Akaike's and Bayesian information criterium (AIC and BIC), discriminative performance (by Harrell's c-statistic, Gönen en Heller's k c-statistic and Chambles C/Cumulative AUC), and risk classification [by Integrated Discrimination Index (IDI)]. All these measures were calculated in ten different imputation sets and results were pooled across sets.

## Results

### Patient Characteristics

We made use of an existing cohort as described in an earlier study (8), and extended this cohort with 57 patients operated in between 2017 and 2019. In total 254 eligible patients underwent awake surgery between 2010 and 2019. We included 109 LGG and 145 HGG patients with a median survival time of 1,490 and 511 days, respectively. Descriptive characteristics (after multiple imputations with ten imputation sets) are presented in Table 2. As expected, most of our determinants significantly differed between HGG and LGG. These results supported the choice for stratified analysis according to tumor grade.

**Table 2:** Baseline characteristics.

**Determinant**	LGG^**^	HGG^**^	***p*-value**
	**Median [IQR]**	**Median [IQR]**	
Total number of patients	109	145	
Tumor-volume (cm^3^)	48.71 [20.71–75.04]	71.14 [28.00–134.50]	<0.001^*^
Age at first surgery	400 [34.00–500]	600 [54.00–67.00]	<0.001^*^
Survival in days	1,490 [694–2554]	5100 [269.00, 774.00]	<0.001^*^
	**N (%)**	**N (%)**	
**WHO2016**			
Grade II/III Astrocytoma IDH-M	62 (56.6)	-	NA
Grade II/III Oligodendroglioma 1p19q deletion	47 (43.4)	-	NA
Grade II/III Astrocytoma IDH-WT	-	15 (10.0)	NA
Glioblastoma IDH-M	-	10 (7.2)	NA
Glioblastoma IDH-WT	-	120 (82.8)	NA
Cognitive impairments			
Executive functioning and attention (−2)	15 (13.7)	53 (36.8)	<0.001^*^
Memory (−2)	4 (4.0)	55 (37.5)	<0.001^*^
Psychomotor speed (−2)	10 (9.0)	45 (30.7)	<0.001^*^
Visuospatial functioning (−2)	9 (8.6)	34 (23.1)	0.010^*^
Language (−2)	4 (4.0)	32 (21.8)	<0.001^*^
Executive functioning and attention (−1.5)	34 (31.3)	83 (57.0)	<0.001^*^
Memory (−1.5)	22 (20.0)	86 (58.8)	<0.001^*^
Psychomotor speed (−1.5)	15 (14.1)	58 (39.9)	<0.001^*^
Visuospatial functioning (−1.5)	15 (14.1)	54 (37.2)	0.001^*^
Language (−1.5)	10 (9.3)	51 (35.3)	<0.001^*^
**Extent of resection**			<0.001^*^
1–78 %	63 (57.5)	35 (23.9)	
79–90 %	23 (21.4)	39 (26.5)	
91–100 %	23 (21.1)	72 (49.6)	
Midline crossing	36 (33.4)	60 (41.4)	0.403
MGMT-methylation	NA	72 (49.4)	NA
Neurologic deficits at presentation	71 (65.5)	112 (76.9)	0.071
Karnofsky performance score (≥70)	105 (96.8)	126 (86.6)	0.015^*^
Seizures at presentation	79 (72.6)	77 (53.3)	0.004^*^
Sex (female)	39 (35.5)	50 (34.7)	0.957
**Location (measured on T2 FLAIR)**			
Frontal	87 (79.9)	107 (73.6)	0.340
Temporal	45 (41.3)	83 (57.3)	0.020^*^
Parietal	31 (28.5)	82 (56.1)	<0.001^*^
Occipital	9 (8.3)	32 (21.9)	0.010^*^
**Hemisphere**			0.075
Left	63 (58.0)	101 (69.4)	
Right	39 (35.9)	36 (24.8)	
Both	5 (5.2)	8 (5.8)	

*P-value refers to the difference between HGG and LGG. IQR, interquartile range; LGG, low grade glioma; HGG, high grade glioma*

* *p < 0.05 (threshold significant value)*.

** *Low grade regarding to WHO 2016 criteria. Grade II/III Astrocytoma IDH-mutated, Grade II/III Oligodendroglioma 1p19q deletion. High-grade: Grade II/III Astrocytoma IDH-Wildtype, Glioblastoma IDH-mutated and IDH-Wildtype. Variables do not always add up to the total number of patients, because the average of 10 imputation sets has been taken*.

For the domains executive functioning and memory, 2% of data was missing. Visuospatial functioning had the highest percentage of missing values of all cognitive domains with 14.2%. In the extent of resection, 15.7% of values were missing, while 20% of values in midline-crossing (only for LGG) and 33.7% of MGMT-status (only for HGG). All the other variables had missing values between 1–6.3%. Supplementary Table 1 shows that patients without and with one or more missing values differed in terms of baseline characteristics, meaning data were not MCAR. Therefore, missing data were accounted for using multiple imputations.

### Neuropsychological Data and Survival

Cognitive impairments (*Z*-values ≤ −2) in HGG, were most common for the domain memory and executive functioning (37.5 and 36.8%, respectively). In LGG, wherein we used thresholds of −1.5 *SD*, deficits were 31.3% for executive functioning and 20% for domain memory.

The univariable survival analyses for all five cognitive domains and other variables are shown in Supplementary Table 2 (stratified by grade). We did not find collinearity between KPS and cognition.

### Hazard Assumptions, Influential Cases, and Functional Form of Prognostic Factors

The PH assumption was checked for all determinants with log-minus-log plots and by Schoenfeld residuals and was found to hold for all variables.

We calculated Df-beta residuals to estimate for each patient by how much the β estimate for each prognostic factor would change if that patient was deleted from our database. In HGG patients there were no influential cases. For LGG we found two influential patients (with change in B-coefficients > 0.5). We checked why these patients were of such great influence. Both patients died early while having prognostic favorable determinants (1p19q deletion, extent of resection >91%, no cognitive impairments). We decided not to exclude these patients, because of the risk for a data-driven model.

Because of a non-linear relation between pre-operative tumor volume and survival in HGG patients and age and survival in LGG patients, we changed the functional form of these variables. “Tumor volume” was log-transformed to “log tumor volume” (in HGG models) and “Age in years” was squared to (Age-42.3) (2), with 42.3 being the mean age in our study population. We had to exclude KPS in the LGG models because of the lack of variability (almost all patients had KPS of 70 or higher). In both patient groups, we merged the “biopsy” and “1–78% resection” categories in the “extent of resection” predictor, because of low frequencies in the “biopsy” category.

### Added Values and Multivariable Models

The results of added value assessments for all different cognitive domains in both patient groups are shown in Tables 3, 4. Only the cognitive domain memory showed significant prognostic value in addition to the established, pre-selected predictors in HGG patients. Loglikelihood of the model without cognition showed a value of −4303 (df = 9) vs. −428.9 (df = 10) for the model with memory included (likelihood ratio test *p*-value = 0.018). The cumulative AUC for HGG with memory included was 0.78 (*SD* = 0.017) and without cognition 0.77 (*SD* = 0.018). Integrated discrimination index (IDI) was 0.043 (0.000–0.102).

**Table 3:** Mean added prognostic value for each cognitive domain in high-grade glioma.

**Cognitive domain (Z-value−2 or lower)**	**No cognition included in model**	**Memory**	**Executive functioning**	**Psychomotorspeed**	**Visuospatial functioning**	**Language**
**Risk classification**						
1. IDI (95% CI) 2. NRI (continuous) (95% CI)	***NA*** ***NA***	0.043 (0.000–0.102) 0.301 (−0.035–0.477)	0.003 (−0.015–0.047) 0.158 (−0.139–0.340)	0.001 (−0.004–0.026) 0.034 (−0.204–0.235)	(−0.011–0.030) 0.090 (−0.241–0.289)	(−0.004–0.029) 0.040 (−0.211–0.266)
**Discrimination**						
1. Harrell's c-statistic (SD) 2. Gönen and Heller's c-statistic (SD) 3. Cumulative AUC (Chambles C) (SD)	0.72 (0.013) 0.71 (0.015) 0.77 (0.018)	0.73 (0.011) 0.72 (0.014) 0.78 (0.017)	0.72 (0.015) 0.71 (0.016) 0.77 (0.020)	0.72 (0.013) 0.71 (0.014) 0.77 (0.018)	0.71 (0.014) 0.71 (0.015) 0.77 (0.018)	0.72 (0.013) 0.72 (0.014) 0.77 (0.018)
AIC BIC	882.06 906.56	877.80 905.03	880.89 908.12	882.99 910.22	883.26 910.49	882.71 909.94
**LL and LLR test** LL with df Chi^2^ *p-*value	−4303 (df = 9) ***NA***	−428.90 (df = 10) 5.638 0.018	−430.44 (df = 10) 1.902 0.171	−431.49 (df = 10) 0.464 0.497	−431.63 (df = 10) 0.401 0.527	−431.34 (df=10) 0.936 0.334

*LLR test is cognition model vs. no cognition included in model, based on pooled chi-square (over 10 imputation sets). LL, loglikelihood; LLR, loglikelihood ratio; IDI, integrated discrimination index; NRI, net reclassification index; AIC, akaike information criterion; BIC, bayesion information criterion; AUC, area under the curve; df, degrees of freedom.

**Table 4:** Mean added prognostic value for each cognitive domain in low-grade glioma.

**Cognitive domain (Z-value−2 or lower)**	**No cognition included in model**	**Memory**	**Executive functioning**	**Psychomotorspeed**	**Visuospatial functioning**	**Language**
**Risk classification**						
1. IDI (95% CI) 2. NRI (continuous) (95% CI)	***NA*** ***NA***	0.063 (−0.012–0.201) 0.359 (−0.178–0.637)	0.027 (−0.029–0.196) 0.185 (−0.366–0.582)	0.047 (−0.007–0.174) 0.149 (−0.236–0.590)	0.013 (−0.018–0.108) −0.049 (−0.422–0.456)	0.002 (−0.009–0.152) 0.068 (−0.535–0.484)
**Discrimination**						
1. Harrell's c-statistic (SD) 2. Gönen and Hellers c-statistic (SD) 3. Cumulative AUC (Chambles C) (SD)	0.85 (0.022) 0.82 (0.016) 0.86 (0.019)	0.87 (0.015) 0.83 (0.014) 0.89 (0.015)	0.86 (0.026) 0.84 (0.024) 0.88 (0.025)	0.87 (0.017) 0.82 (0.018) 0.88 (0.019)	0.85 (0.023) 0.82 (0.016) 0.88 (0.021)	0.85 (0.022) 0.83 (0.017) 0.87 (0.020)
AIC BIC	110.27 115.88	109.19 115.60	109.19 115.61	109.31 115.72	111.50 117.92	111.63 118.05
**LL and LLR test** LL with df Chi^2^ *p*-value	−48.13 (df = 7) ***NA***	−46.59 (df = 8) 2.338 0.127	−46.60 (df = 8) 1.997 0.160	−46.65 (df = 8) 2.456 0.118	−47.75 (df = 8) 0.610 0.435	−47.82 (df = 8) 0.388 0.534

*LLR test is cognition model vs. no cognition included in model, based on pooled chi-square (over 10 imputation sets). LL, loglikelihood; LLR, loglikelihood ratio; IDI, integrated discrimination index; NRI, net reclassification index; AIC, akaike information criterion; BIC, bayesion information criterion; AUC, area under the curve; df, degrees of freedom.

**Figure 1:**
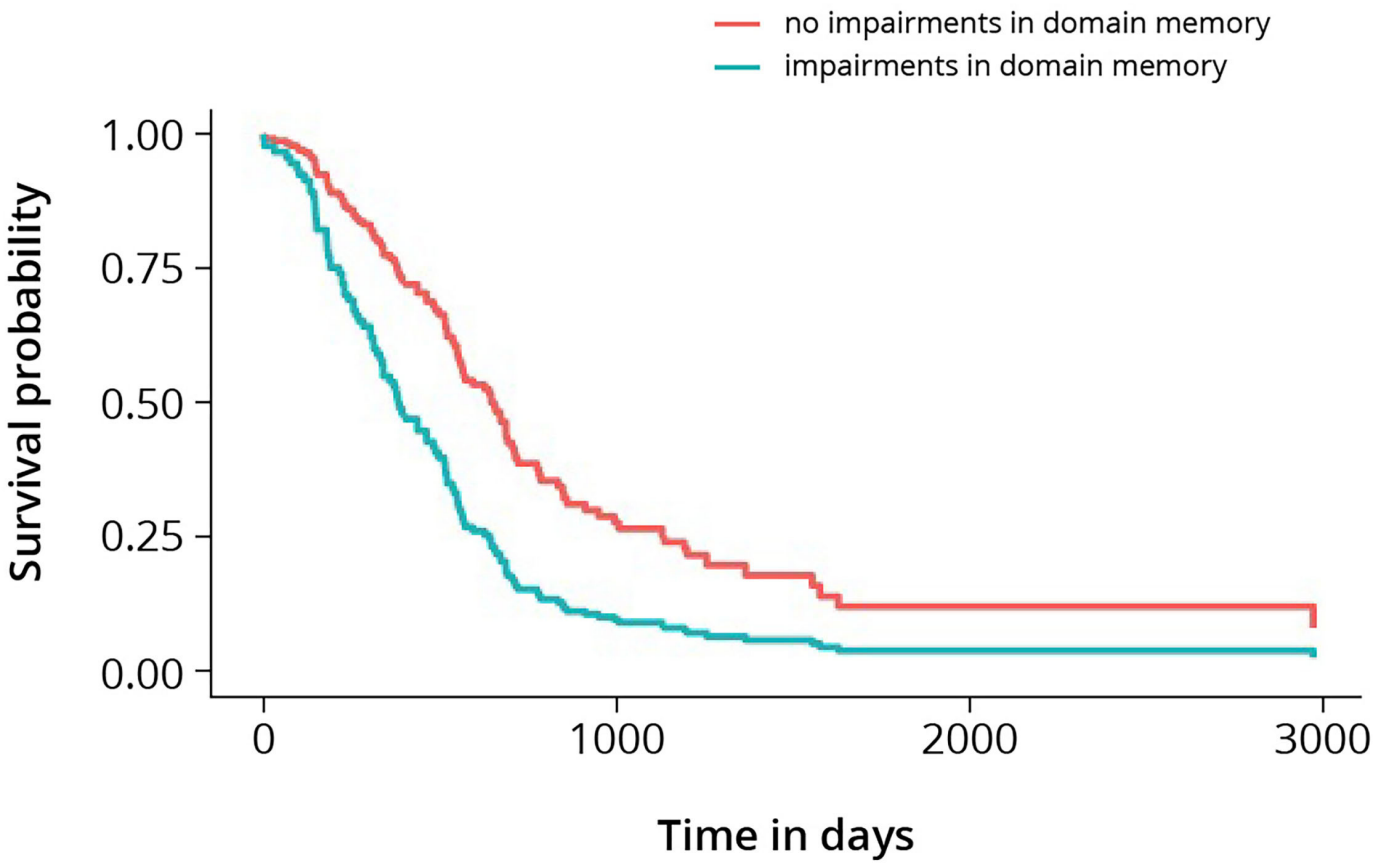
Cumulative survival curves for high-grade glioma, stratified by memory. Log-rank test shows *p*-value < 0.005.

The multivariable model with memory included is presented in Table 5. Impairments in memory showed a significant association with survival [hazard ratio = 1.71 (*p*-value = 0.018; CI; 1.1–2.63)] in presence of the pre-selected predictors age at presentation, the extent of resection, neurologic deficits, epileptic seizures, KPS, WHO-2016 classification, and pre-operative tumor volume. In Figure 1, cumulative survival curves for this model are shown, stratified by memory performance.

**Table 5:** Multivariable cox-regression model with memory included in high-grade glioma.

**Variable**	**HR**	**Lower 95% CI**	**Upper 95% CI**	**Estimate B**	**Std-Error (SE)**	***p-*value**
**Memory**	1.706	1.104	2.635	0.534	0.222	0.018^**^
**Extent of resection**						
(1–80%=ref)						
81–90 % 91–100 %	0.517 0.557	0.265 0.302	011 029	−0.659 −0.585	0.342 0.313	0.061^*^ 0.070^*^
**WHO−2016**						
Grade II/III–WT=ref Grade IV IDH-Mut Grade IV IDH-WT	092 3.169	0.281 002	4.240 10.029	0.088 1.154	0.692 0.588	0.899 0.057^*^
**Seizures at presentation**	050	0.611	1.804	0.049	0.276	0.860
**MGMT/methylation**	0.491	0.274	0.878	−0.712	0.297	0.024^**^
**KPS (1–69=ref)** **70–100**	0.501	0.239	049	−0.692	0.377	0.077^*^
**LogVolume**	039	0.820	1.318	0.038	0.121	0.753
**Age at presentation**	046	021	070	0.045	0.012	0.0003^**^

*HR, Hazard ratio*.

* *p-value < 0.1*

** *p-value < 0.05. Ref, reference category*.

In LGG, none of the cognitive domains was of added prognostic value, the results of the model without cognition included are shown in Supplementary Table 3.

## Discussion

The goal of this study was to investigate the prognostic value of cognitive functioning in treatment-naive patients with diffuse gliomas (low grade and high grade), in addition to well-recognized predictors of survival in these patients. In the multivariable Cox-regression model with HGG, the cognitive domain memory had significant prognostic value when added to a model which included molecular subtype, MGMT-methylation, the extent of resection, age at diagnosis, KPS, seizures at presentation, and tumor volume. In other words, the prognosis of a patient could be predicted more precisely if memory deficits are included as a predictor in prognostic models for overall survival in HGG. In LGG, we did not find the additional value of any of the five cognitive domains.

In earlier work, we already showed that cognitive deficits are independently associated with survival (8). However, the focus of this recently published study was etiologic, rather than prognostic. This means that the main goal was to demonstrate the independent, and possibly causal, the relationship between cognitive deficits and survival at a group level. This was in contrast with the aim of this study, which was prognostic and took place at an individual level with the aim of estimating the risk for an individual patient. Following from these two distinct yet related study designs, it was a logical step to take the findings from the previous etiological study and investigate the added prognostic value of these previously identified factors in the current prognostic study, for their predictive value at the individual level. As a consequence of the difference in focus, we performed different types of statistical analyses in both studies. Additionally, we included different determinants in the models than in the etiologic study. For the present study, all possible predictors were extracted from prediction models previously published in the literature. Finally, in this current study, we conducted separate analyses in two different study subpopulations (LGG and HGG).

As a result of differences in analyses, we found noticeable differences in results as well. We did not find executive functioning to be of significant added value in prediction models. Apparently, executive functioning has an insufficient predictive value at the individual patient level. According to the literature, our results were domain-specific and memory was more strongly correlated with survival than other cognitive domains in HGG (7, 8). Hypothetically, memory is more vulnerable to the effects of the structural nuance of the infiltrative tumor and metabolic changes in the tumor environment (33–35). Cognitive functioning in this domain may be hampered before more structural changes occur and therefore may reflect the aggressiveness of the tumor in a more sensitive way than MRI. In LGG we did not find additional prognostic value of any of the five cognitive domains. However, the predictive performance of the model without cognition included was already high (Harrell's c-statistic = 0.85), which makes it more difficult to demonstrate the added value of a predictor. This is also known as a “ceiling effect”. If a model already predicts the data almost perfectly, the chance becomes smaller that makes new variables in your model add significant value.

A second explanation can be that cognitive impairments are less common in LGG and as a consequence the lower threshold used to define ‘impairment' in LGG. These factors, combined with the low number of events in this subgroup, cause insufficient power to establish a relationship between survival and cognition. A third explanation for the difference in the added value of cognition between subgroups is that various glioma subtypes differ greatly in their biological behavior as well as their prognosis. Possibly, the effect of cognition – and interaction with underlying pathophysiological mechanisms of the tumor – differs between WHO 2016 glioma subtypes.

We included variables for the HGG prediction models based on the most validated and recently published nomograms. We used most elements from the nomogram of Gorlia et al. but used WHO2016 classification instead of WHO2007 for tumor grade and histomolecular classification (2). Additionally, we focused on domain-specific neuropsychological assessment instead of Mini-Mental State Examination (MMSE) as a measure for cognitive functioning. We confirmed the published prognostic value of extensive cognitive testing, age at presentation, and MGMT-methylation status. At a more liberal threshold (*p* ≤ 0.1) as is admitted in prognostic research; WHO2016 classification, KPS, and extent of resection were of predictive value as well. The fact that the extent of resection was not significantly correlated, stresses the need to assess this extent or resection with volumetric methods rather than surgical reports.

We found that 53.3% of HGG patients, while 72.6% LGG, presented with seizures as their first symptom. Presentation with seizures has traditionally been identified as an independent positive prognostic factor (26, 36). The observed prognostic effect might result from distinct biological features of epileptogenic tumors (26). Our study did not find a presentation with seizures to be a statistically significant prognostic factor (*P* = 0.86). However, the strong prognostic effect has been demonstrated particularly in GBM, IDH-WT tumors, and our subgroup of HGG included grade IV IDH-mutated tumors as well (26). We did not remove the variable 'epilepsy at presentation' from the final model as we prespecified the variables we wanted to include, to avoid data-driven results and overfitting of our model (37). Tumor size has been described in the literature as an important prognostic factor as well, independent of tumor grade. We did not find tumor size to be a statistically prognostic factor in our model (*p* = 0.753). A possible explanation for this could be the way tumor volume was measured (based on a very liberal FLAIR volume which could have underestimated the relation with survival). Additionally, in literature tumor size is an independent prognostic factor of tumor grade, but the grade is based on WHO-2007 classification (1, 2). Hypothetically, WHO-2016 predicts survival better than WHO2007 classification and therefore tumor size becomes redundant in our model (38). Again, because described in the literature as a well-known prognostic factor, we kept this factor in our model.

For the model of LGG, we included variables based on different nomograms to be as complete as possible (1, 3, 5). The recently published nomogram for LGG patient survival by Gittleman et al. included tumor grade, molecular subtype, KPS, age at diagnosis, and sex. In the well-known prediction model of Gorlia et al. presence of neurologic deficits and tumor size are also included. Midline crossing, age at presentation, and WHO2016 classification were significant predictors in our multivariable model. Unfortunately, we had to exclude KPS from our model, because the frequencies of patients with KPS ≤ 70 were too low. The presence of neurologic deficits was frequent at presentation (65.6%) but did not correlate to survival in our model; neither did the extent of resection and sex. This may be related to the composition of our study population and difference with other study populations, wherein non-awake operated patients were included too. In earlier work, we described the differences between awake and non-awake operated patients (7). In general, patients in our cohort were relatively young and had good performance status. Also, the proportion of oligodendrogliomas was higher. Furthermore, the reason why we did not find a relation between the extent of resection and survival may also be related to the fact that we based the degree of resection on the surgical report. The variability of this factor might be less reproducible in this way. Another possibility is that extent of resection is already influenced by cognitive monitoring during operation, which could have reduced the prognostic value of this determinant.

Rather than measuring cognitive changes postoperatively, pre-operative cognitive functioning was used to determine the impact of cognition on survival. Cognitive functioning at baseline represents the unbiased effects of the tumor on the underlying brain networks best, as cognitive functioning during follow-up can be influenced by surgical procedure and postoperative treatment as well. From a practical point of view, informing patients about their prognosis is most valuable in the earliest stages of the disease, when treatment choices have yet to be made. For the same reason, we did not include post-operative treatment in any of our models; post-operative treatment is not known at the moment of diagnosis yet. Since WHO-2016 diagnosis and extent of resection are included in the model, it is applicable during the early postoperative timeframe, when the medical team discusses the results from histological analysis with a patient. Prognostic data are most useful at this time, for patient counseling and as an aid for patients and physicians in therapeutic decision-making.

Our study has several strengths. In other studies, cognitive testing often consisted of MMSE or other cognitive screening tools instead of extensive domain-specific testing (2, 39, 40). We used comprehensive methods to establish the added value of cognitive functioning, based on the most recent recommendations (32). Further strengths of our study are the relatively large HGG sample size, the standardized NCF testing prior to surgical resection, the conservative cut-off value of *Z*-values for cognitive impairments (which adds further to the robustness of our findings), and the significant proportion of patients with tumor involvement of the right hemisphere, as opposed to many cognition-aimed studies in glioma, with an overrepresentation of left hemisphere-tumors.

Limitations of our study should also be mentioned. At our center, NCF was routinely performed in patients undergoing awake surgery, which carries the risk of selection bias. As published before (7), these patients may have different characteristics than those undergoing biopsy or standard resection. In addition, the percentage of LGG patients is higher in the group of awake surgery patients than in the total glioma population (7). However, since we included all consecutive patients that underwent awake surgery, regardless of their cognitive performance or their outcome (survival), we feel that our analyses offer a valid description of the relation between cognitive performance and survival, without selection bias and without compromising the internal validity of our study. Still, it is possible that this selection of patients has influenced the generalizability (external validity) of our results.

Another factor that could have led to selection bias is the selective loss to follow-up of patients who had insufficient neuropsychological data to perform analyses on. The reason for having insufficient data was often emergency surgery in case of rapid clinical decline. This could have led to exclusion of patients with cognitive impairments and worse clinical performance and therefore we possibly underestimated the relation between cognitive functioning and survival. Finally, we decided to group tasks on their conceptual background (“domain”) to enhance power; analyses per task would add up to an undesirable number of analyses and could potentially obscure findings for the overarching cognitive domain. The question of which cognitive concept (or domain) is best represented by a specific task is always complicated since intrinsically more than one concept is tapped in any task. However, neuropsychologists do share common ground in the categorization of tasks across domains, and we grouped tasks according to such shared insights (24, 25). Finally, due to missing data, we had to use multiple imputation methods. However, missing data were considered to be 'missing at random' and cognitive domains had low frequencies of missing data. Lastly, we used advanced imputation methods with multiple imputation sets, which minimizes the risk of bias due to missing data, and data between these different sets did not differ significantly.

## Conclusion

Our findings supported the hypothesis that the pre-operative presence of memory deficits, as measured with detailed neuropsychological assessment (NPA), was of additional prognostic value in high-grade glioma when added to other well-known predictors of overall survival. This finding was domain-specific and was not found in low-grade glioma.

Ultimately, parts of the NPA could be implemented in prognostic models for glioma patients. In the full, extensive form, neuropsychological testing may not be practical to implement in prediction models, so a shorter NPA should first have to be developed, containing those tests with the highest predictive value.

## Data Availability Statement

The raw data supporting the conclusions of this article will be made available by the authors, without undue reservation.

## Ethics Statement

The studies involving human participants were reviewed and approved by Institutional Review Board (or Ethics Committee) of University Medical Center of Utrecht (protocol code METC 17/384 and 09-420 on 2nd of august 2018). Written informed consent for participation was not required for this study in accordance with the national legislation and the institutional requirements.

## Author Contributions

EvK and TJS: Conceptualization and supervision. EvK, IHW, CR, FDV, JV, TS, and MvZ: Data curation. EvK and ES: Formal analysis. PR: Funding acquisition and investigation. EvK, ES, and TJS: Methodology. EvK: Writing–original draft. ES, IHW, CR, FDV, JV, TS, MvZ, PR, and TJS: Writing–review and editing. All authors contributed to the article and approved the submitted version.

## Funding

This research was funded by Ton & Patricia Bohnenn Fund for Neuro-oncology.

## Conflict of Interest

The authors declare that the research was conducted in the absence of any commercial or financial relationships that could be construed as a potential conflict of interest.

## Publisher's Note

All claims expressed in this article are solely those of the authors and do not necessarily represent those of their affiliated organizations, or those of the publisher, the editors and the reviewers. Any product that may be evaluated in this article, or claim that may be made by its manufacturer, is not guaranteed or endorsed by the publisher.
