# Supplementary material for: Added Value of Cognition in the Prediction of Survival in Low and High Grade Glioma
Source: Front Neurol. 2021 Nov 18;12:773908. doi: 10.3389/fneur.2021.773908 (PMC8639204; doi:10.3389/fneur.2021.773908)
Supplement: Supplementary file 1 [file Table_1.DOCX]

| **Determinant**  **Supplementary table 1:** Baseline characteristics organized by patients who had missing data in any variable or without missing data. SE = standard error of the mean. | **With missing data** | **Without missing data** | **p-value** |
| --- | --- | --- | --- |
|  |  |  |  |
|  | **Mean [SE]** | **Mean [SE]** |  |
| Total number of patients | 226 | 8 |  |
| Tumor-volume (cm^3^) | 74.80 (4.09) | 74.68 (18.09) | 0.995 |
| Survival in days | 1120.50 (71.66) | 710.88 (183.27) | 0.306 |
|  |  |  |  |
|  |  |  |  |
|  | **N (% of row)** | **N (% of row)** |  |
| WHO2016 |  |  | 0.069 |
| *Grade II/III Astrocytoma IDH-M* | 58 (100) | 0 (0) |  |
| *Grade II/III Oligodendroglioma 1p19q deletion* | 43 (100) | 0 (0) |  |
| *Grade II/III Astrocytoma IDH-WT* | 13 (100) | 0 (0) |  |
| *Glioblastoma IDH-M* | 6 (100) | 0 (0) |  |
| *Glioblastoma IDH-WT* | 106 (93.0) | 8 (7) |  |
| Cognitive impairments |  |  |  |
| *Executive functioning and attention (-2)* | 62 (93.9) | 4 (6.1) | 0.128 |
| *Memory (-2)* | 56 (96.6) | 2 (3.4) | 0.908 |
| *Psychomotor speed (-2)* | 48 (92.3) | 4 (7.7) | 0.048 |
| *Visuospatial functioning (-2)* | 32 (94.1) | 2 (5.9) | 0.455 |
| *Language (-2)* | 33 (94.3) | 2 (5.7) | 0.375 |
| Extent of resection |  |  | 0.605 |
| *1-78 %* | 81 (97.6) | 2 (2.4) |  |
| *79-90 %* | 49 (94.2) | 3 (5.8) |  |
| *91-100 %* | 76 (96.2) | 3 (3.7) |  |
| Midline crossing | 19 (65) | 1 (5.0) | 0.794 |
| MGMT-methylation | 35 (89.7) | 4 (10.3) | 0.781 |
| Neurologic deficits at presentation (=yes) | 176 (96.2) | 7 (3.8) | 0.322 |
| Karnofsky performance score (≥70) | 215 (96.4) | 8 (3.6) | 0.456 |
| Seizures at presentation (=yes) | 152 (98.1) | 3 (1.9) | 0.147 |
|  |  |  |  |
| Location (measured on T2 FLAIR) |  |  |  |
| *Frontal* | 187 (96.9) | 6 (3.1) | 0.914 |
| *Temporal* | 122 (96.1) | 5 (3.9) | 0.487 |
| *Parietal* | 107 (96.4) | 4 (3.6) | 0.730 |
| *Occipital* | 39 (97.5) | 1 (2.5) | 0.791 |
| Hemisphere |  |  | 0.877 |
| *Left* | 158 (96.3) | 6 (3.7) |  |
| *Right* | 73 (97.3) | 2 (2.7) |  |
| *Both* | 14 (100) | 0 (0) |  |
