# Supplementary material for: Added Value of Cognition in the Prediction of Survival in Low and High Grade Glioma
Source: Front Neurol. 2021 Nov 18;12:773908. doi: 10.3389/fneur.2021.773908 (PMC8639204; doi:10.3389/fneur.2021.773908)
Supplement: Supplementary file 2 [file Table_2.DOCX]

| Supplementary table 2: Baseline characteristics in high grade glioma and low grade glioma. Crude (unadjusted) relation between both the five cognitive domains of interest and other determinants and survival. P-value refers to results of univariable analyses per subgroup  *=p-value<0.05. **Low grade regarding to WHO 2016 criteria. Grade II/III Astrocytoma IDH-mutated, Grade II/III Oligodendroglioma 1p19q deletion. High-grade: Grade II/III Astrocytoma IDH-Wildtype, Glioblastoma IDH-mutated and IDH-Wildtype. | | | | | | | | |
| --- | --- | --- | --- | --- | --- | --- | --- | --- |
|  | **Alive or Censored**  **HGG**** | **Event**  **HGG**** | **Hazard-rate**  **HGG**** | **p-value**  **HGG**** | **Alive or Censored**  **LGG**** | **Event**  **LGG**** | **Hazard-rate**  **LGG**** | **p-value**  **LGG**** |
|  | **Median [IQR]** | **Median [IQR]** |  |  | **Median [IQR]** | **Median [IQR]** |  |  |
| Total number of patients | 33 | 113 |  |  | 92 | 17 |  |  |
| Tumor-volume (cm^3^) | 59.60  [27.07-105.76] | 77.04  [28.00-136.47] | 1.09 | 0.27 | 35.16  [20.24-68.83] | 69.61  [30.73-88.20] | 1.008 | 0.11 |
| Age at first surgery | 53.00 [46- 62] | 64 [58- 70] | 1.05 | <0.001* | 41.00 [35-52] | 43.00 [29-59] | 1.02 | 0.48 |
| Survival in days | 726 [311-1210] | 480 [228-685] | NA | NA | 1617  [714-2624] | 1243  [547- 2033] | NA | NA |
|  | **N (%)** | **N (%)** |  |  |  |  |  |  |
| WHO2016 |  | | | | | | | |
| Grade II/III Astrocytoma IDH-M | 0 (0.0) | 0 (0.0) | NA | NA | 47 (51.1) | 15 (88.2) | ref | ref |
| Grade II/III Oligodendroglioma 1p19q deletion | 0 (0.0) | 0 (0.0) | NA | NA | 45 (48.8) | 2 (13.3) | 0.18 | <0.001* |
| Grade II/III Astrocytoma IDH-WT | 8 (25.3) | 6 (5.6) | Ref | Ref | 0 (0.0) | 0 (0.0) | NA | NA |
| Glioblastoma IDH-M | 3 (7.6) | 8 (7.0) | 1.77 | 0.29 | 0 (0.0) | 0 (0.0) | NA | NA |
| Glioblastoma IDH-WT | 22 (67.1) | 98 (87.4) | 5.47 | <0.001* | 0 (0.0) | 0 (0.0) | NA | NA |
| Cognitive impairments |  | | | | | | | |
| Executive functioning and attention (-2) | 6 (16.8) | 48 (42.6) | 1.72 | 0.005* | 12 (12.9) | 3 (18.2) | 2.31 | 0.42 |
| Memory (-2) = 1 | 7 (22.3) | 47 (42.0) | 2.20 | <0.001* | 3 (3.4) | 1 (7.9) | 2.31 | 0.42 |
| Psychomotor speed (-2) | 7 (20.7) | 40 (33.6) | 1.47 | 0.05* | 7 (8.0) | 2 (14.5) | 1.27 | 0.76 |
| Visuospatial functioning (-2) | 7 (20.1) | 27 (23.9) | 1.19 | 0.41 | 7 (7.9) | 2 (12.1) | 1.02 | 0.98 |
| Language (-2) | 7 (22.6) | 24 (21.6) | 1.33 | 0.22 | 4 (4.7) | 0 (0.0) | NA | NA |
| Executive functioning and attention (-1.5) | 12 (36.3) | 71 (63.0) | 1.81 | 0.003* | 30 (32.5) | 4 (24.2) | 1.27 | 0.69 |
| Memory (-1.5) | 13(39.9) | 72 (64.4) | 1.94 | <0.001* | 17 (18.7) | 5 (27.3) | 1.65 | 0.35 |
| Psychomotor speed  (-1.5) | 10 (30.5) | 48 (42.7) | 1.56 | 0.02* | 11 (11.8) | 4 (26.7) | 2.30 | 0.15 |
| Visuospatial functioning (-1.5) | 10 (30.2) | 44 (39.3) | 1.17 | 0.42 | 11 (12.3) | 4 (24.2) | 1.45 | 0.52 |
| Language (-1.5) | 10 (30.8) | 41 (36.6) | 1.21 | 0.32 | 9 (9.9) | 10 (6.1) | 0.71 | 0.75 |
| Extent of resection |  | | | | | | | |
| 1-80 % | 7 (20.7) | 28 (24.8) | ref | ref | 53 (57.2) | 10 (59.4) | ref | ref |
| 81-90 % | 9 (28) | 29 (26.0) | 0.55 | 0.02 * | 19 (20.5) | 4 (26.7) | 1.39 | 0.55 |
| 91-100 % | 17 (51.2) | 55 (49.2) | 0.61 | 0.04* | 21 (22.3) | 2 (13.9) | 1.09 | 0.90 |
| Midline crossing | 5 (14.6) | 55 (49.2) | 1.18 | 0.39 | 27 (29.2) | 94 (57.0) | 2.77 | 0.04* |
| MGMT-methylation | 19 (56.4) | 53 (47.4) | 0.50 | <0.001* | NA | NA | NA | NA |
| Neurologic deficits at presentation | 27 (82.0) | 85 (75.5) | 0.97 | 0.89 | 59 (64.1) | 12 (73.3) | 1.32 | 0.60 |
| Karnofsky performance score (>70) | 29 (88.7) | 97 (86.0) | 0.68 | 0.18 | 89 (96.6) | 16 (97.6) | NA | NA |
| Seizures at presentation | 18 (54.3) | 60 (53.0) | 0.93 | 0.71 | 70 (76.1) | 16 (97.6) | 0.38 | 0.05* |
| Sex (female) | 16 (50.0) | 34 (30.2) | 0.72 | 0.11 | 36 (38.6) | 3 (18.2) | 0.30 | 0.07* |
| Location (measured on T2 FLAIR) |  | | | | | | | |
| Frontal | 26 (78.0) | 81 (72.4) | 1.25 | 0.31 | 73 (79.6) | 13 (81.2) | 0.89 | 0.85 |
| Temporal | 17 (50.3) | 67 (59.3) | 1.10 | 0.71 | 40 (41.8) | 6 (38.8) | 1.05 | 0.92 |
| Parietal | 20 (61.0) | 62 (54.7) | 1.10 | 0.77 | 24 (26.0) | 7 (42.4) | 2.02 | 0.16 |
| Occipital | 8 (24.4) | 24 (21.1) | 0.86 | 0.51 | 7 (7.6) | 2 (12.1) | 1.51 | 0.59 |
| Hemisphere |  | | | | | | | |
| Left | 20 (62.2) | 80 (71.6) | ref | ref | 51 (54.9) | 13 (75.8) | ref | ref |
| Right | 10 (30.5) | 26 (23.1) | 0.99 | 0.97 | 36 (39.0) | 3 (18.2) | 0.40 | 1.16 |
| Both | 3 (7.3) | 6 (5.3) | 2.20 | 0.07 | 5 (5.0) | 1 (6.1) | 1.36 | 0.77 |
