# Supplementary material for: Added Value of Cognition in the Prediction of Survival in Low and High Grade Glioma
Source: Front Neurol. 2021 Nov 18;12:773908. doi: 10.3389/fneur.2021.773908 (PMC8639204; doi:10.3389/fneur.2021.773908)
Supplement: Supplementary file 3 [file Table_3.DOCX]

**Supplementary table 3: Multivariable cox-regression model with no cognition included in low-grade glioma.** HR= Hazard ratio. *=p-value < 0.1 **=p-value< 0.05. Ref = reference category. CI = confidence interval.

| Variable | HR | Lower 95% CI | Upper 95% CI | Estimate B | std.error (SE) | p.value |
| --- | --- | --- | --- | --- | --- | --- |
| **Extent of resection**  (1-80%=ref)  81-90 %  91-100 % | 1.711  0.476 | 0.340  0.053 | 8.603  4.277 | 0.537  -0.742 | 0.824  1.120 | 0.536  0.536 |
| **WHO-2016**  Grade II/III Astrocytoma IDH-M=ref Grade II/III Oligodendroglioma 1p19q deletion | 0.092 | 0.014 | 0.613 | -2.386 | 0.968 | 0.049* |
| **Midline crossing** | 9.873 | 2.172 | 44.883 | 2.290 | 0.773 | 0.021* |
| **Sex** | 0.350 | 0.080 | 1.528 | -1.049 | 0.751 | 0.202 |
| **Age [age-43]^2^** | 1.003 | 1.0008 | 1.005 | 0.003 | 0.001 | 0.033* |
| **Neurologic deficits** | 1.757 | 0.027 | 5.028 | 0.162 | 0.741 | 0.834 |
